# Evidence gaps in ENT surgery – a qualitative survey

**DOI:** 10.3205/cto000137

**Published:** 2016-12-15

**Authors:** Jan Löhler, B. Akcicek, F. Müller, G. Dreier, J. J. Meerpohl, W. Vach, J. A. Werner

**Affiliations:** 1 German Study Center of Oto-Rhino-Laryngology, Head and Neck Surgery, Bonn, Germany; 2 Scientific Institute for Applied Otolaryngology, Bad Bramstedt, Germany; 3 University Department of Otolaryngology, Philipps University of Marburg, Germany; 4 Study Center of the University Hospital of Freiburg, Germany; 5 Cochrane Germany, University Hospital of Freiburg, Germany; 6 Department of Medical Biometry and Medical Informatics, University Hospital of Freiburg, Germany

**Keywords:** evidence-based medicine, ENT medicine, evidence gaps, survey, healthcare research

## Abstract

**Introduction:** As in other disciplines, the burgeoning knowledge in ENT medicine long ago surpassed our ability to adequately absorb it and maintain a proper overview. This can give rise to actual or assumed evidence gaps that can impede the progress of the discipline and evidence-based treatment of patients. Clinics and medical practices also hold to traditional doctrines that shape day-to-day medicine, without these schools being challenged based on evidence.

**Methods:** Between February and June 2015, 160 ENT clinics, including 34 university hospitals, and 2,670 ENT practices took part in a two-arm online survey on existing or perceived evidentiary gaps in ENT medicine using a previously developed questionnaire. The survey used for half of the participants was open in form; the other half were given a closed survey with systematics of the field for orientation. The survey was augmented with additional data such as the number of publications and focus areas in the clinics and the age and type of practice of the established physicians.

**Results:** The return rate from the clinics was 39.7%; the return rate of the closed surveys was 29.3%. Of the physicians in medical practice, 14.6% responded to the closed and 18.6% to the open survey. There were no major differences between the two forms of survey. Otological and oncological issues comprised approximately 30% of the list of answers from clinics. Corresponding questions were formulated regarding the current diagnostic and therapeutic problems, such as with stage-related tumor treatment or implantable hearing aids. Diagnostic procedures, e.g., special new procedures in audiology and vestibulogy, dominated the surveys from the practices. However clinics and practices alike cited marginal areas of the discipline that are of daily relevance.

**Discussion:** The cited evidence gaps then needed to be verified or refuted and clarified based on research of the literature as to whether the existing evidence actually reached healthcare providers in the form of guidelines, publications, conferences, or continuing training for application in daily practice. Other steps would include prioritizing future research, evidence mapping, deciding on further systematic reviews, and targeted studies in conjunction with procuring third-party funding and in cooperation with patient associations. The knowledge thus gained should ultimately be transferred in improved form for application in daily clinical practice. Ten questions of key importance each needed to be formulated for the hospitals and practices.

## 1 Introduction

For clinical routine, evidence-based medicine becomes more and more important [1]. The current knowledge in every discipline is enormous – and so also in ENT. Every day numerous new original publications are added internationally. Review articles provide an overview about the current status of research, however, there is the risk that a certain selection of the used original publications causes a weighting of the overall conclusion of such narrative review articles [2]. Another methodical approach is pursued by systematic review articles. The formulation of questions according to a particular scheme, exactly defining the patients (P) or populations, interventions (I), comparable intervention (C = control), and endpoints (O = outcomes), i.e. the so-called PICO questions help assessing systematically all relevant primary trials that may be included by precisely mentioning the inclusion criteria. This leads to a lower risk for a systematic bias compared to the subjective literature research for narrative review articles. Such systematic reviews (SR) usually encompass the following 5 steps:

1. formulating a question,
2. systematic research of the literature,
3. evaluation of the quality,
4. summary and if appropriate statistical synthesis,
5. interpretation.

SR systematically assess and summarize medical procedures. If an SR includes several studies with nearly homogenous results, they can be pooled statistically in a meta-analysis; this means that SR can, but not need to, contain meta-analyses. Narrative review articles, however, comprehensively describe a disease with regard to diagnostics and therapy [2], [3], [4].

This flood of information comprises more than 20,000 biomedical journals per year with worldwide more than 1 million of scientific articles and nearly doubles every 10 years [5]. It is actually no longer possible to manage this jungle of medical literature [6]. If a physician had wanted to be up to date in 1993, he would have had to read about 17 original papers per day or at least carefully study one key publication. The actual time spent on literature was about 30 minutes per week on average [7], [8]. In 2013, 11 systematic review articles and 75 primary study were published, until now the numbers are continuously increasing [2], [9].

Those aspects that apply for practically working physicians, are also true for colleagues who actively perform research in order to increase the specific knowledge. In the scientific context, another problem is observed. On the one hand each group focuses on the own field of research and extends the current knowledge together with the international scientific society. But on the other hand, this permanent focus on single areas leads to neglecting neighboring or related subjects because there is no group dealing with this part. Even very large university hospitals cannot dispose of specialists for really every field of otolaryngology. Over the time, real evidence gaps within a discipline may open, white spots on the map of knowledge. Altogether, those problems lead to a lack of research knowledge and activities and also to a lack of specialists regarding treatment.

Certain scientific questions cannot be clarified within one single hospital or group; either because the organizational efforts are too high, the existing resources of staff and material too low, the incidence of certain diseases too rare, or the number of cases that would be necessary to gain reliable statements is too high for one hospital. Ideally, clinical trials should be carried out in a randomized, controlled, and multicenter way. Furthermore, another reason for knowledge gaps or evidence gaps is the aspect of tradition that must not be underestimated. This means that diagnostic and therapeutic procedures that were once introduced are not questioned. Even an evidence gap that is not recognized as such may occur.

So despite the present, daily growing enormous flood of information in our discipline there is a lack of multicenter, controlled (prospective) trials to answer important questions. The consequences may be immense as seen on the example of the former discussions about the benefit of tonsillectomy or the therapy of sudden hearing loss [10], [11], [12], [13].

The method of evidence mapping tries to comprehensively and clearly present the current knowledge on a topic [2]. While systematic review articles follow a generally accepted and clear method, the methodical approach in the context of evidence mapping is not finally consented. Hereby, most relevantly two terms are used, the term of “evidence map” representing a mostly tabular overview of the current status of research with regard to the number of trials, characteristics of the studies, characteristics of the patients, and study results, and the term of “scoping reviews” describing the before-mentioned aspects in a narrative way.

In 2013, a sort of guideline for the methodical procedure to establish an evidence map was developed on the basis of a systematic literature research [2]. The authors recommend the following steps:

1. Prioritization and definition of a question taking into consideration the prevalence, incidence, morbidity, mortality, quality of life, and costs; formulation of this question based on a modified PICO scheme mostly putting aside the control as well as the outcomes but adding criteria for the study design (S) (PI(CO)S scheme).
2. Systematic research of the literature according to the exact question.
3. Selection of trials first based on the title and abstract and then in a second step based on the full text.
4. Tabular data extraction by means of standardized documentation forms.
5. Possible assessment of the quality of the identified literature (only applicable for scoping reviews)
6. Presentation of the results as tables/databases for evidence maps and as descriptively narrative text for scoping reviews.

Based on a broadly formulated question that is mostly not limited with regard to certain controls or outcomes, an extensive overview of the existing trials is provided that indirectly also shows the missing evidence, i.e. the evidence gaps. By identifying white spots regarding a lack of knowledge in the context of clinical studies and systematic reviews, evidence mapping can be used as preparatory work for the identification of certain research areas that have been ignored and for performing systematic reviews. This is important on the one hand for transferring knowledge into the research field because everyone who is interested may have an overview about the existing literature on a certain topic. On the other hand this procedure can be used in the context with other institutions such as political decision makers, cost bearers (e.g. health insurances) in order to present existing evidence in a simple way. With this background, the presence or lack of evidence of controlled multicenter studies may be important from an economic point of view regarding the continuation of our discipline as a whole but also for every hospital department and every single practice. And of course evidence is essential for the treatment of every individual patient.

## 2 German Study Center of Otolaryngology

In November 2012, the presidents of both German ENT-specific organizations, i.e. the German Society of Otolaryngology, Head and Neck Surgery, Bonn, Germany, and the German Professional Association of ENT Specialists, Neumünster, Germany, decided to establish the German Study Center of Otolaryngology, Head and Neck Surgery.

The German Study Center closely cooperates with the German Register of Clinical Trials, which is the national study register for Germany in the network of the WHO-accredited registers [14]. Thus ENT-specific studies become selectively visible. The team of the German Study Center is the contact organization for physicians in private practices and hospitals, it gives advice before starting trials, supports with the calculations of costs, and provides statistical, regulatory, and organizational support. Furthermore, the Germany Study Center supports third party funding, recruiting of study centers, establishing national and international cooperation, writing applications to official authorities and ethical committees, and registering trials. Due to the close connection to the study center of the University of Freiburg, Germany, existing interdisciplinary competence can be used in a targeted way [1]. In this context it is crucial that the Society and the Association pursue the same objectives. It became rapidly obvious that it might be reasonable that the Study Center pursues a systematic approach in order to identify evidence gaps in otolaryngology and to contribute effectively to an improved evidence situation.

## 3 Identification of evidence gaps in ENT

As already described initially, there are absolute and relative evidence gaps regarding different topics despite the multitude of publications. For the future of otolaryngology it is crucial to identify the evidence gaps so that different groups may close them – also supported by the German Study Center of Otolaryngology. However, it is neither reasonable nor possible to perform evidence mapping for the whole discipline. Apart from the immense efforts that would have to be undertaken it would not become clear where the gaps actually are.

Another approach could be to use the knowledge of all ENT specialists about existing or suspected evidence gaps of their own disciplines. In 2013, the Dutch Society of Otolaryngology (*Nederlandse Vereniging voor Keel-Neus-Oorheelkunde en Heelkunde van het Hoofd-Halsgebied, NVKNO*) published a science agenda (*De KNO-Wetenschapsagenda* [15]). In this context, the NVKNO established a group of scientist to inventory existing ENT-specific knowledge and to prioritize it with regard to its relevance in daily routine of Dutch ENT physicians. This project had 3 parts.

1. Overview of the current scientific activities and comparison with neighboring countries.
2. Inventory of the scientific knowledge of ENT physicians.
3. Summary of the results in a science agenda.

For this purpose, a catalogue of questions was sent to all Dutch University ENT departments where the following questions had to be answered:

1. Main fields of research in 2011
2. Number of doctoral theses from 2008 to 2011
3. Titles of those theses

In addition, ENT-specific publications were looked up based on clearly defined entries in scientific databases that were classified according to the type of publication (original paper, review article etc.) and scientific journal. The number of scientific publications and their impact factor in the Netherlands were compared to those from neighboring countries from 2001 to 2011. Based on the same procedure, the ENT guidelines were assessed and evaluated.

The knowledge reported by the ENT physicians was inventoried. Additionally, the existing guidelines and the WiKiNo, a Dutch interactive database of evidence-based knowledge in otolaryngology run by the Dutch ENT Society, were analyzed.

Furthermore, all members of the NVKNO and all relevant patient associations were asked. They were invited to submit the five most important, subjectively perceived evidence gaps of daily routine in the form of research questions and at the same time assign a degree of urgency and relevance. This study was completed by the data of the James Lind Association [16] on the topic of vestibular pathologies.

This organization is an English association of physicians and patients advising and supporting methodically to assess unanswered questions in medicine, to prioritize, and to provide the results of prioritization for further research. For this purpose, the organization publishes a manual on how physicians and patients may cooperate as partners regarding the prioritization of research questions. In the field of audiology, the data of the Dutch program of hearing tests (*Nationaal Programma Gehooronderzoek, NPG*) were completed. This program includes ENT physicians, audiologists, and patients who suggest different topics for possible audiological research in order to improve the situation for people suffering from hearing impairment.

On June 25, 2012, during a scientific meeting with 40 selected ENT specialists and representatives of associations regarding ENT patients, the evidence gaps were assigned to the different ENT related sections and prioritized according to different criteria such as relevance, feasibility of research, and impact on medicine. Beside the prioritization of the research questions, three subgroups were introduced for each section where deficits in basic research, in guidelines, and in the category “others” were described related to realization and control of the aspects. 

This Dutch study was the basis to carry out a survey on evidence gaps in otolaryngology in Germany. Because of the different sizes of both countries and the different national structures of the health care systems (for example there are nearly no private ENT practices in the Netherlands), the survey was modified.

The objective of this investigation was to identify real and suspected evidence gaps in otolaryngology in Germany by asking possibly all ENT departments and practices. Beside the absolute assessment of evidence gaps as well as a possibly different perception in practices and hospitals, we wanted to find out if the answering behavior was influenced by a pre-defined closed scheme on otolaryngology. Additionally, side parameters should be assessed, e.g. the type of practice or if it was a department of a university hospital. In contrast to the NVKNO we did not ask patient associations nor perform a research of literature databases in terms of publications, guidelines, or study registers. It is planned to investigate these aspects in a second step when the evidence gaps are verified or falsified as well as open research questions are prioritized, and also when sponsors, cost bearers, and other stakeholders are included. Only the number of the different expected answers from hospital departments and practices was already much higher than from the Dutch colleagues. In Germany, there are much more than 8 ENT departments in university hospitals, in addition to general hospitals and thousands of ENT practices.

## 4 Methods

### 4.1 Systematics of otolaryngology and questionnaires

#### 4.1.1 Systematics of otolaryngology

First of all, the main authors of this study elaborated a system of the ENT discipline that describes most exactly and exhaustively all different sections regarding diagnostics and therapy. In this context, deliberately certain frequently applied methods were explicitly mentioned and others were summarized in special categories.

Of course, there are overlaps between the categories, e.g. some aspects of laryngology are also relevant for the section of pediatric ENT or ENT oncology, and allergic diseases are also relevant for diseases of the nose and the paranasal sinuses etc. So the mentioned categories cannot have a clearly separating but classifying function. In order to avoid unnecessary doublings, typing was only made in one category. The elaborated list does not claim to be complete. 

In most of the categories, the methods of direct and optic examination for diagnosis were not mentioned even if mirror examination and endoscopy still play a major role in all areas of the ENT discipline; this is mainly also true for imaging (ultrasound, CT scan/CBT, angiography, MRI, PET etc.).

This ENT-specific system is found in Table 1 (Tab. 1). It was also used as basis for the classification of the answers.

The survey among the departments and practices was conceived in a two-arm design. In an open arm the below-mentioned questions were asked without additional information, in the closed arm, the mentioned system was provided for further orientation (Table 1 (Tab. 1)). This system should give an orientation for answering the questionnaires on current evidence and evidence gaps in the field of otolaryngology. This classification together with the information that own and other areas could be indicated was communicated to the institutions of the closed survey.

#### 4.1.2 Questionnaires for hospitals

The following questionnaire (see section 5) developed by the first author of this study was the basis of the online survey for hospitals. The second part of the first sentence (in brackets) was missing in the open survey, apart from that, the forms were identical.

## 5 Questionnaire on evidence and evidence gaps in otolaryngology

Please indicate the fields and questions as exactly as possible (please use the attached classification system). Please indicate the fields that seem relevant to you personally.

1. In your opinion, which three areas of otolaryngology currently require particular research?
  a. …
  b. …
  c. …
2. In your opinion, which three evidence gaps are currently especially important?
  a. …
  b. …
  c. …
3. In your opinion, which three specific questions should be answered urgently?
  a. …
  b. …
  c. …

1. Does your department belong to a university hospital?
  a. Yes
  b. No
2. What are currently the most important fields of research of your institution (max. 3)?
  a. …
  b. …
  c. …
3. How many publications (original papers) were published by you and your co-workers in 2013 and 2014?

### 5.1 Questionnaire for medical practices

The following questionnaire developed by the principal authors of this study was the base for the online survey of practices. The last sentence (in brackets) was missing in the open survey, apart from that, the forms were identical.

## 6 Questionnaire for improvement of the treatment of ENT-specific patients

Please indicate the fields and questions as exactly as possible. Please only indicate the areas and questions that are relevant according to your experience. If there are less than three relevant aspects, please mention only those. (Please use the attached classification system.)

1. Please write down three diagnostic methods of otolaryngology where in your opinion the usefulness of diagnosis, advice, and therapeutic decision is most uncertain.
  a. …
  b. …
  c. …
2. Please write down three therapeutic options in otolaryngology where in your opinion the benefit for the patient is most uncertain.
  a. …
  b. …
  c. …
3. Which three means would you consider desirable to support decisions and therapy for your patients? Those might be apps for their smartphones (e.g. a reminder of appointments for specific immunotherapy, for treatment of hearing impaired patients etc.). Or there might be brochures, documents, information where you can define certain objectives that seem to be reasonable or important to achieve and optimize the healing process (e.g. way of life, nutrition in the context of sleep apnea or carcinoma).
  a. …
  b. …
  c. …

1. Which type of practice are you working in?
  a. Single practice
  b. Joint practice (every physician should fill out a questionnaire)
2. Which additional specialization do you have?

1. How old are you?
2. Please indicate your gender.

### 6.1 Process of survey

In order to keep the administrative efforts as low as possible, the hospitals and practices were contacted via e-mail. This e-mail contained an explanation of the project and the hospitals and practices were provided with an internet link (https://de.research.net/s/..., at the end of this address the single questionnaires were linked) that led to the online form. According to their affiliation, the participants had to answer the above-mentioned questions. This procedure limited the number of the contacted practices because not all ENT practices dispose of an e-mail address.

The e-mails for the hospitals were sent out by the office of the German ENT Society at Bonn, for the practices it was carried out by the office of the Professional Association of Otolaryngologists at Neumünster. The assignment to the closed or open survey was randomly performed.

The letter to the hospitals and practices was identical apart from the last survey of the hospitals (see section 7, annexes 1 and 2).

**Hospitals:** A total of 160 heads of ENT departments were contacted, among them 34 university professors and 126 chief physicians in general hospitals. The closed survey encompassed 16 university professors and 62 chief physicians, i.e. 78 departments. The open survey was sent to 18 university professors and 64 chief physicians, i.e. 82 departments. 

**ENT practices:** A total of 2,670 ENT practices were contacted. The closed survey was sent to 1,336 physicians, the open survey encompassed 1,364 physicians.

The process of questioning is shown in Table 2 (Tab. 2). Besides the primary sending of mails, Table 2 (Tab. 2) contains the dates of sending out reminders. Furthermore, the group of university professors and chief physicians were informed personally about the project at the occasion of the 86^th^ Annual Meeting of the German Society of Otolaryngology, Head and Neck Surgery. The database was closed in the evening of June 15, 2015. This database had been established by the Study Center of Freiburg. Each participant of the survey had a personal ID number.

## 7 Coding of data

All data entered up to June 15, 2015, were transferred into 4 Excel files, one each for the replies from the open and closed surveys of hospitals and practices. For further processing, those data were translated into numeric codes. The attempt was made to assign all the answers, also those from the open survey, to the classification system of the closed survey in order to better compare the groups. In certain cases, this was not possible (see below). For single groups and questions specific modifications had to be performed that will be described more in detail. The primary evaluation tables had nearly 150 pages.

### 7.1 Coding of question 1 (questionnaire for hospitals)

For coding the first question about the three fields of otolaryngology where research is currently mostly required sent to hospitals of the closed survey, the mentioned classification system was used. A numeric seven-digit code was established assigning a two-digit value to the main topics (otology, rhinology etc.) beginning with 10 (Table 3 (Tab. 3)). Using the value of 01 for the first item, the computer system would have created a six-digit code because of the 0. The other values code the sub-classifications (Table 3 (Tab. 3)). If no sub-items or sub-specialties were explicitly mentioned in certain areas, 0 was added until 7 digits were achieved.

Regarding question 1 of the open survey of hospitals, a new 4-digit coding scheme was developed where a 2-digit value starting with 10 was assigned to the main topics. The answers from this area were very individual. In order to assess the information as exactly as possible, this classification system had more exact main items that were initially conceived as sub-items so that this classification had only 4 digits despite further sub-classifications. An example for this procedure is the newly established main item of “inner ear” that in the 7-digit code of the closed survey of hospitals and of the ENT-specific classification was a sub-item of the category “otology”. In summary, a comparative presentation of the answers of the closed question 1 could be achieved. Additionally, it was coded if the hospitals of the closed survey observed the pre-defined classification (value 1) or not (value 2).

### 7.2 Coding of question 2 (questionnaire for hospitals)

The coding of the second question to hospitals about the three most relevant evidence gaps was carried out based on two different schemes. According to the general, above-mentioned, 7-digit numeric system a scheme was developed including single items that were mentioned frequently such as sinusitis and nasal polyposis that were assigned to the new item of “single diseases” in the category of “rhinology”. The open survey was coded based on the systematic classification described in Table 1 (Tab. 1). The 7-digit code was not applied since the 4-digit one based on the answers allowed a higher precision. By including new sub-items and avoiding redundant coding options, finally a higher exactness was achieved. Additionally, it was coded if the hospitals of the closed survey observed the pre-defined classification (value 1) or not (value 2).

### 7.3 Coding of question 3 (questionnaire for hospitals)

The third question to hospitals about the three most urgent scientific issues required a scheme that was completely adapted to the answers. For this purpose, the answers were systematically numbered with a 2-digit number starting with 10 that in a second step was assigned to the according topic.

### 7.4 Coding of additional questions (questionnaire for hospitals)

The affiliation of the single departments to a university hospital was coded with the value 1 for existing affiliation and value 2 for not existing affiliation. The research focus was coded according to the third question, the answers were assessed individually, numbered with a 2-digit value starting with 10, and then assigned to the according topic.

### 7.5 Coding of question 1 (questionnaire for practices)

The first question to ENT practices about the diagnostic methods with most uncertain benefit for diagnosis was coded based on the 7-digit scheme of ENT-specific items (Table 1 (Tab. 1)) that was already described for question 1 to hospitals. This coding was applied to the open and the closed survey. Additionally, it was coded if the practices of the closed survey observed the pre-defined classification (value 1) or not (value 2).

### 7.6 Coding of question 2 (questionnaire for practices)

Regarding the second question about the three therapeutic options with the most uncertain benefit for the patient, the classification of Table 1 (Tab. 1) was applied in a modified way, for example the newly developed item of “therapy” was completed by explicit therapeutic options such as phytotherapy. Additionally, it was coded if the practices of the closed survey observed the pre-defined classification (value 1) or not (value 2).

### 7.7 Coding of question 3 (questionnaire for practices)

The 7-digit coding of the answers of the third questions about desirable tools for decision making and therapy support was created freely based on the classification system of Table 1 (Tab. 1). Different groups with single sub-groups were elaborated that reflect the answers as exactly as possible.

### 7.8 Coding of additional questions (questionnaire for practices)

Regarding the type of practice, different codes were assigned (single practice = 1, joint practice = 2). The question about the additional specializations was coded in a 2-digit way. Answers comprising more additional specializations were summarized in groups with 2, 3, 4, or more with and according numeric code.

### 7.9 Other codings

Regarding questions requiring numeric answers such as the number of publications in 2013 and 2014 (hospitals) or the age of the physicians (practices) were not coded in a particular way. The mentioned value was taken.

Answers that could not be clearly assigned were coded with a value consisting of 8, adapted to the number of digits of the single codes (8888888, 8888). For question where no answer was given, a code consisting of 9 was used adapted to the number of digits of the single codes (9999999, 9999).

## 8 Data evaluation

The collected data were evaluated by means of descriptive statistics. Hereby, absolute and relative frequencies were calculated and summarized in a table. The analysis was performed with the program “Statistical Analysis System (SAS), version 9.2”. 

The generated tables were manually assembled for every question and sometimes newly assigned. Hereby, primarily the defined scheme for the closed survey (Table 1 (Tab. 1)) was applied. The items that were included in the sense of higher exactness were also manually added. In some areas, the sub-items were assigned to the general items in an aggregating way because the individual description did not provide new information. Regarding the third question to practices, only the general items were described for this present contribution (see below).

## 9 Results

In the evening of June 15, 2015, the database contained the following replies:

- Hospitals (closed survey): 31, 14 of them were university hospitals (mailing to 78/16)
- Hospitals (open survey): 24, 9 of them were university hospitals (mailing to 82/18)
- Practices (closed survey): 195 (mailing to 1336)
- Practices (open survey): 248 (mailing to 1334)

**Figure 1:**
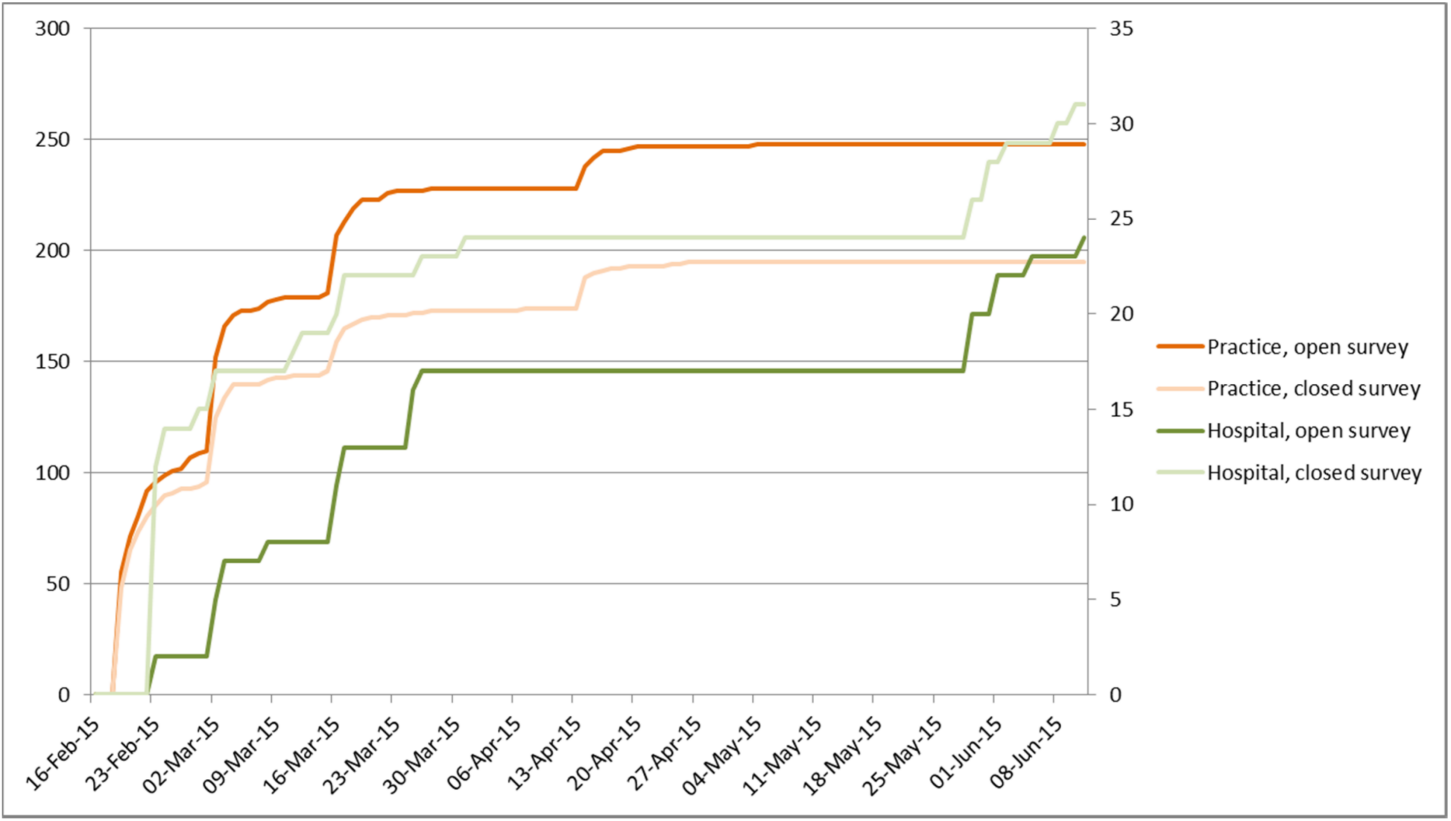
Time accumulation of the returned questionnaires (Table 2). Hospitals: right ordinate axis; practices: left ordinate axis.

Figure 1 (Fig. 1) shows the time accumulation of the number of returned surveys depending on the mailings described in Table 2 (Tab. 2). The statistical details about the participants of the survey are found in Table 4 (Tab. 4), Table 5 (Tab. 5), Table 6 (Tab. 6).

The results encompass nearly 150 pages with different single tables. It would go far beyond the scope of this contribution to describe everything in detail. In the following, a selection of the results will be presented that seem to be most relevant regarding the primary questions of this study. In case that some topics are not described even if they were explicitly mentioned beforehand, it is because they occurred with a lower incidence than others. Furthermore, the accumulation of similar answers should contribute to a higher relevance of the questions asked in this study. Subsequent publications will emphasize more details.

### 9.1 Results regarding hospitals

The answers to the three key questions to hospitals are summarized in Table 7 (Tab. 7), Table 8 (Tab. 8), Table 9 (Tab. 9).

The fields with particular need of research (question 1) are illustrated in Table 7 (Tab. 7). The currently most important evidence gaps (question 2) are found in Table 8 (Tab. 8). Results on questions that urgently need investigation (question 3) are listed in Table 9 (Tab. 9).

The answers from the closed and open survey on the research focus of hospitals are depicted in Table 10 (Tab. 10), data on publications in 2013 and 2014 are found in Table 11 (Tab. 11). The median number of publications of the closed survey was 6 compared to 9 in the context of the open survey. The overall median value was also 9. Six of 9 university hospitals (66.7%) and 4 of 14 general hospitals (28.6%) had a number of publications above the median value. The median of the number of publications for university hospitals was 15 compared to 4.5 of other hospitals per year.

### 9.2 Results regarding practices

The answers of the 3 key questions to practices are summarized in Table 12 (Tab. 12), Table 13 (Tab. 13), Table 14 (Tab. 14).

The fields that are characterized by particular diagnostic uncertainty (question 1) are listed in Table 12 (Tab. 12). Areas where the therapeutic benefit is uncertain (question 2) are presented in Table 13 (Tab. 13).

The results of question 3 regarding desirable tools for daily practice already originally encompass 10 pages. Since their content is of high relevance for daily routine but has only subordinate importance for the detection of evidence gaps in otolaryngology, a complete list is not given here. Table 14 (Tab. 14) shows the titles of the single categories and mentions single examples to complete the overview.

## 10 Discussion

### 10.1 Basic data and response rates

The response rate was highest for the closed survey of hospitals, especially of university hospitals. Around 90% of the university professors returned the questionnaires. This is the only noticeable difference between the groups. However, this higher percentage of the responses of the closed survey in comparison to the open survey is relativized if the small absolute number of both groups is taken into account. The difference of the response rate of hospitals and practices is significant (p<0.0001), however irrelevant regarding its importance (see below). Even the group of chief physicians returning the questionnaires of the closed survey is a bit higher compared to the open survey.

The type of survey had no significant impact (p = 0.62) comparing the open with the closed survey in an isolated way. However, a relationship can be found considering the interactions between the type of the institution (hospital or practice) and the type of survey (open or closed). The following odds-ratios can be found (p = 0.031):

- Hospital vs. practice
  - Closed survey: 3.86 (95% confidence interval: 2.39; 6.23)
  - Open survey: 1.81 (95% confidence interval: 1.10; 2.97)
- Closed vs. open survey
  - Hospitals: 1.59 (95% confidence interval: 0.83; 3.08)
  - Practices: 0.75 (95% confidence interval: 0.61; 0.92)

Regarding the observance of the suggested ENT-specific classification system, the picture is not uniform. While nearly 60% of the closed survey of hospital directors observed the pre-defined classification regarding question 1 (areas with particular need for research), only about 25% observed this system when answering question 2 (currently most relevant evidence gaps).

In the context of the practices, the response rate was similar regarding the percentages in both arms (about 17%); the practices of the open survey was a bit bigger compared to the closed survey. Because of the significantly higher number of practices, the absolute number of responses was higher compared to the hospitals even if they had a clearly higher response rate regarding the percentage. Figure 1 (Fig. 1) shows that every reminder mailing led to a further increase of the number of responses. The isolated increase for the hospitals in June 2015 was due to the fact that the university professors and chief physicians were informed explicitly during the annual meeting of the German ENT Society in 2015. Those increases of the responses are typical [17], [18], [19], [20], [21], [22]. According to the literature, generally a higher response rate can be expected in short digitalized surveys [19], [23].

In general, the response rates of surveys among physicians have a high variability because of different reasons [24]. The average value are rates of about 54% [25], but the rates are continuously decreasing since many years [26]. Low response rates may potentially lead to a lower validity of the statements. This is especially possible for the practices of the survey. The average age of the physicians in practices was about 51 years ranging from 30 to 75. There were no considerable differences regarding the survey arms of the practices. The 1^st^ and 3^rd^ quartile encompassed an age of 46 to 56 years each. The comparable structure of both groups allows drawing the conclusion that there were no differences between both groups of the physicians from practices that participated in the survey. Since the average age of all physicians in Germany amounts to 53.41 years [27], which is nearly exactly the same as for physicians who participated in our survey with about 51 years, a possible non-responder bias can be excluded in our study [24], [28].

Around 32% of the physicians of practices were females which is congruent with the percentage of female colleagues in practices [27]. Nearly 40% are working in joint practices.

## 11 Hospitals

### 11.1 Question 1: fields with currently urgent need of research

Regarding the answer to the question to hospitals about field with currently urgent need for research, general topics were mentioned, a detailed description or classification was not provided. In both survey arms (closed and open), oncology ranks first, followed by otology and rhinology (Table 7 (Tab. 7)). It is noticeable that in the arm of the open survey, pediatric otolaryngology and the field of diseases of the joints are not mentioned. While the last-mentioned fact concerns diseases that directly touch neighboring disciplines, it is astonishing with regard to the intensive discussion of tonsillectomy and adenoidectomy [10], [11] of the last years that those areas were probably not that present in the group of the open survey than in the arm of the closed survey. However, in a synopsis with the next questions, this phenomenon is put into a perspective.

### 11.2 Question 2: currently very important evidence gaps

In contrast to question 1, Table 8 (Tab. 8) lists the more detailed statements of both groups that partly orient with the systematic classification or that can be easily included. Also here, the three main topics of oncology, otology, and rhinology rank first. In the closed survey, the participants gave more detailed answers than the group of the open survey. Especially in the field of oncology, an exact description of the areas is given (e.g. the effectiveness of surgery vs. radiochemotherapy, biomarkers, antibodies, sensitivity). In the group of the open survey, generally prospective studies are encouraged. In the field of otology, both groups mention the pathogenesis and therapy of inner ear diseases as areas of important evidence gaps. It is interesting that in the open survey the single entities of sudden hearing loss and tinnitus were mentioned several times whereas those diseases were not stated in the closed survey. In the field of vestibulogy and audiology as sub-areas of otology, instrument-based examinations, in particular posturography and adaptive speech-hearing tests were given as significant evidence gaps in the closed survey. In the field or rhinology, both groups focus on chronic rhinosinusitis regarding important evidence gaps. Other areas with evidence gaps are comparable to question 1, the indication of tonsillectomy, the treatment of swallowing disorders, sleep medicine as well as diseases of the thyroid gland. Furthermore, general therapy studies including possible late effects of systemic therapies are requested.

### 11.3 Question 3: questions with urgent need for answers

In this context, the application of the ENT classification system was not intended so that both arms of the survey were not compared. It is remarkable that the answers given for question 2 have gained further significance in the cumulative assessment. In first place were again the three main topics of oncology, otology, and rhinology, additionally issues of daily routine were mentioned that cannot be discussed individually in the frame of this paper because of the multitude of answers and the limited space. The topics listed in Table 9 (Tab. 9) were classified into further sub-items (e.g. definition, prevalence, diagnostics, therapy etc.). Beside the already mentioned questions from the field of pediatric otolaryngology, even others on therapeutic efficiency in allergology, on sleep medicine, on diagnostics (PET vs. CT scan) up to special questions on preparation of ENT-specific instruments and basic questions (delay of dementia by preserving the ENT related senses). The authors invite the readers to reflect about the items listed in Table 9 (Tab. 9). Important impulses may result for the future of our discipline by approaching them with concerted efforts (see below).

## 12 Additional questions (hospitals)

### 12.1 Focus of research

Regarding the research focuses of hospitals, oncology and otology were mentioned as main fields in both groups. In oncology, topics concerning tumor biology, diagnostic and therapeutic procedures as well as prognostic factors were mentioned. Interesting and for the development of our discipline certainly important is the fact that also skin tumors and rare tumors were listed as research focuses. For a better overview, the field of otology was subdivided into a general part (diagnostics and therapy) and a special part. In the special part, clinically most relevant diseases are found such as sudden hearing loss, tinnitus, and Menière’s disease but also the question of maturation of the auditory system and relatively frequent issues that result from functional disorders of the auditory tube. Unsurprisingly, the surgical hearing prosthesis was often mentioned as research focus. Also in this context, interesting prospective questions are asked as for example the relationship between cochlear implantation and the impact on the sense of balance.

In rhinology, again questions on surgery of the paranasal sinuses were mostly mentioned. The example of rhinology shows impressively how certain areas overlap. Some question concern the area of sleep medicine and allergology and vice versa. As described initially, a table can only describe approximately such a complex intertwining reality. The same is true for the described questions on dysphagia and the quality of life of cancer patients. Comparable to question 2, the diseases of the salivary glands takes relatively little space. Regarding their number, also areas that are methodologically challenging are rather small. Those concern questions on biomarkers, immunotoxins, stem cell therapy, and sensory neurobiology that touch and overlap with basic research.

### 12.2 Publications 

At a first glance, the number of publications listed in Table 11 (Tab. 11) seems to reveal an imbalance between the groups of the closed and the open survey. However, this is probably artificial. The median of the number of publications in both groups was about 7 papers per year per institution (closed survey: 6; open survey: 9). The spectrum of the numbers was very large, it ranged from 0 to 110 original papers between 2013 and 2014. Since both groups had only small case numbers, those individual variations in the distribution with random effects can be explained. However, considering the number of institutions that are above the group median according to their affiliation to a university or not, 13 of 14 and 7 of 9 university hospitals, respectively, were above this median which is due to the research assignment (and possibilities) of university professors.

## 13 Medical practices

### 13.1 Question 1: fields with particular diagnostic uncertainties

General differences between the closed and the open survey were not found in the group of the practices regarding the first question. The first item in the list of responses (Table 12 (Tab. 12)) is the field of otology as mostly mentioned item. In this context, especially newly introduced methods of vestibulogy, cVEMP, oVEMP, vHIT, and diagnostic positional exercises for the three semicircular canals were mentioned as fields of particular diagnostic uncertainty. In audiology, the objective procedures of BERA (ABBR, ASSR), the derivatives of DPOAE (growth function, high resolution DPOAE), questionnaires (APHAB) and the adaptive speech and hearing tests that require further clarification according to the colleagues working in practices.

This reflects the current diagnostic development of our discipline, every newly introduced method is naturally associated with open questions. But the study reveals that those questions are present in the group of colleagues working in practices.

Otology is followed by rhinology, also in this context, the current developments are found. So CBT is mentioned as alternative method to CT scan. But also procedures that are associated with open questions for a long time such as rhinomanometry, olfactometry, and gustometry were listed.

Other areas with diagnostic uncertainty are phoniatrics (voice range profile), diseases of the ENT-related joints, methods of allergy diagnosis, diagnostics of salivary gland function, sleep medicine, and diagnostics of the thyroid glands. The issues regarding the diagnostics of head and maxillary joints and the thyroid and parathyroid glands show that also in this area the current developments of our discipline are reflected. Considering the age distribution of the responding colleagues, the possible conclusion might be drawn that the majority of them was not confronted with those methods during their own specialization in that way that is common today. But this is not the case. Applying the chi-square test regarding a possible correlation to this specific question and the age of the colleagues working in practices, the following p values result regarding the diagnostics and therapy of those questions and an age limit of 50 years (median age of the colleagues working in practices: 51 years). A correlation can thus be excluded.

- Thyroid and parathyroid glands: 0.65
- Head and maxillary joints: 0.42
- VEMP (cVEMP and oVEMP): 0.88
- HIT (including vHIT): 0.11

Generally appropriate training programs would be a possible solution to close the mentioned evidence gaps. 

In contrast to the clinical environment, oncology plays only a role regarding diagnostic procedures and staging for the colleagues in practices which can be explained by the fact that patients with suspected malignomas are generally immediately transferred to an ENT department in a hospital for further diagnostics and therapy.

### 13.2 Question 2: fields with particular therapeutic uncertainties

Comparable to the first question, there are no relevant differences in the responses between the cohorts of the closed and open survey. In analogy to question 1, otology ranks in first place of the responses (Table 13 (Tab. 13)). In the context of conservative therapies, mostly methods of habituation (hearing and vertigo training) were mentioned. Regarding the surgical methods, implantable hearing aids and prosthesis are listed which is in analogy to the fields of necessary research of the hospitals.

Also in oncology, the gaps mentioned by the hospitals were listed by the practices (surgical methods, radiochemotherapy, antibody therapy, HPV) even if it was not to the same extent because of the daily routine. The same applies for rhinology and surgical measures. The causal questions of chronic sinusitis are not focused in the same way by the practices – which might be due to the differently formulated questions. However, also the practices mentioned the current therapeutic questions, especially in the context of allergology (SIT vs. SLIT, specific antibodies, ASS). The same is true for sleep medicine where the still open questions of surgery of the velum and the base of tongue were mentioned as well as pediatric otolaryngology with the indications for tonsillectomy and tonsillotomy.

Interestingly, the colleagues in practices of the open survey mention the treatment methods of speech therapy and phytotherapy. Probably there is a need of scientific knowledge transfer.

### 13.3 Question 3: desirable tools for daily routine in practices

As already mentioned in the chapter of results, this question revealed a large number of sometimes very detailed suggestions. They documented a high need of the physicians in practices for support in their daily routine. There were no differences between both groups and because of the openly formulated question 3 in both cohorts they were not expected. In first place, there was information material for patients on different topics regarding therapy, postoperative care, offers but also health care politics and fees in the outpatient sector. Besides, possibility of new media are listed (smartphone apps) that also support communication and information. Finally instruments for knowledge provision and support in the organization of the practice are desired (Table 14 (Tab. 14)). The results of question 3 were completely published in the *HNO-Mitteilungen* of the Professional Association of German Otolaryngologists.

## 14 Summary and outlook

The two arm design allowed applying the strengths of an open and a closed survey at the same time. With the catalogue, a closed survey invites to intensively reflect the questions asked. For example, in the context of the second question where currently the most important evidence gaps are, the topics of diseases of the joints, pediatric otolaryngology, and laryngology were mentioned only in the closed survey. An open survey benefits from the creativity of the responders. Taking the same example of question 2 to hospitals, areas were mentioned that were outside the applied classification system: implantations, therapy studies, and the late effects of systemic (oncologic) therapies. No relevant field of otolaryngology was excluded in the two survey arms. The absolute number of responses of the practices was higher compared to the hospitals, but the percentage of responses of the hospitals was clearly higher. Regarding the practices, it is a representative sample of responses, in the context of the hospitals, it is in some areas nearly an exhaustive survey.

In summary, the result represents an inventory of our discipline. The hospitals reported about diseases and therapies that are currently in the focus of intensive research while the practices described the actual uncertainties of new procedures regarding diagnostics and therapy.

The aim of this study to elaborate the white spots on the map of our discipline was achieved. Naturally, the focus of the hospitals was placed more on surgical procedures (e.g. oncology, cochlear implantation), while the practices were mainly focused on diagnostic procedures (e.g. audiology, oto-neurology). However, this observation only shows two sides of the same coin and emphasized the intertwining division of work of both areas. Question 3 was answered by the hospitals with high exactness whereas practices replied more exactly to the questions 1 and 2. Regarding the classic aspects of our discipline, a high rate was expected from the hospitals (oncology and otology: about 30%; rhinology: about 15%; pediatric otolaryngology: about 15%). The same is true for practices (otology: about 45%; rhinology: about 18% etc.). Furthermore peripheral areas of our discipline such as diagnosis and therapy of thyroid and parathyroid diseases, diseases of the head and maxillary joints were considered as being important for the development of otolaryngology. Together with the interest in speech therapy and phytotherapy this would be an opportunity for both ENT-specific associations to support their members explicitly in training and research.

## 15 Differences compared to KNO Wetenschapsagenda

Comparing our study with the Dutch investigation [15], the following differences must be mentioned. In summary, the Dutch colleagues reported about the same problematic fields. Also there, the current questions on stage-depending cancer therapy (surgery, radiochemotherapy, influence of HPV), implantable hearing aids as well as audiology and vestibular diagnostics ranked first in the lists of the responses. In this context, our survey represents an external verification of the Dutch study.

In the Netherlands, there are only 8 university hospitals. There are neither municipal hospitals with ENT departments, nor are there relevant numbers of ENT practices. At least, they are not mentioned in the investigation of our neighbors. So the high publication rate in relation to the number of inhabitants is not surprising.

In contrast to our survey, the Dutch publication mentions every reply individually. This would not have been possible in our investigation due to the high number of participants. Because of the missing aggregation it would further not have led to a higher gain in knowledge. Interestingly, not all Dutch centers replied to every topic. The response rates were between 7 (oncology, otology) and 1 reply (allergology, sleep medicine). The distribution of the response rates were similar to ours regarding the absolute numbers.

In contrast to the Dutch colleagues, we did not include patient representatives in our survey and did not perform a systematic research of the literature. Both actions would have gone far beyond the scope of our investigation. Furthermore, we had a different intention. The necessary research of the literature can only be performed based on the knowledge gained here in order to achieve a prioritization, to initiate evidence mapping, systematic reviews, and clinical trials. The deviations of both investigations reflect less the differences of the results but rather the relative size of both countries, the differences of the health care systems, and the different stage in the process of identifying evidence gaps in otolaryngology.

## 16 Possible options

This study has a descriptive character. The following steps and recommendations of scientific, organizational, and political actions can be deducted from the results:

**a)** Verification and falsification of the mentioned evidence gaps by according research in order to clarify if the existing evidence is really transported to the physicians by guidelines, publications, meetings, and trainings.

**b)** Prioritization of further research by establishing an agenda. This could be performed in the sense of a ranking by the German ENT Society, the Association of German Otolaryngologists, the German ENT Study Center, and if desired other expert panels. Hereby, an extended benefit analysis [29] or an Analytic Hierarchy Process [30] could be used for better decision making.

**c)** Evidence mapping as described above is useful for those cases where actual evidence gaps are present. Additionally, a difference must be made between assumed and real evidence gaps.

**d)** Decision for further systematic reviews, targeted trials, and further research using existing potentials of hospitals and practices should be made based on those criteria.

**e)** Fund raising (public sponsors, statutory health services, industry).

**f)** Improved transfer into practices, especially by using new media (eHealth, smartphone apps etc.).

Since it is nearly impossible to answer every single question concretely and rapidly, the authors suggest to develop a ten-point plan for hospitals and practices each with regard to the results described in this paper. This plan should include the questions and evidence gaps that require urgent answers. The presidencies of both ENT-related societies as well as the German ENT Study Center should then discuss all these proposals involving other interested colleagues and issue recommendations how those ten-point plans modified after discussion could be pursued in the context of concrete research projects in the sense of the above-mentioned measures and who should be responsible.

Coordinated research with combined resources from hospitals and practices and a systematic knowledge transfer may further develop our discipline and thus better meet the current and future challenges and optimally use the financial means for the benefit of our patients.

Furthermore, it is also important according to the results of this study to further develop our discipline especially in the area of diagnosis and therapy of diseases of the thyroid and parathyroid glands as well as diseases of the joints of the head, the cervical spine and the maxilla.

## 17 Annex

### 17.1 Annex 1: cover letter of the survey

Dear Colleague, 

enclosed please find an important survey for research of evidence gaps in otolaryngology. Based on this survey, we want to find out where we may scientifically best support your daily work in the future.

So we kindly ask you to answer these three very short questions on this topic. Please use the attached classification of our discipline as well as the link mentioned in this letter to participate in this study.

In case of questions, please contact me directly. Thank you in advance.

Sincerely,

Dr. med. Jan Löhler 

Vice-Chairman of the Steering Group of the German ENT Study Center 

German ENT Study Center 

Friedrich-Wilhelm-Str. 2 

53113 Bonn

### 17.2 Annex 2: cover letter of the final survey (hospitals)

Dear Colleagues, 

in the context of the Annual Meeting of our Society in Berlin, Prof. Werner drew your attention again to the importance of the enclosed survey and kindly asked you for your participation. 

With this letter we send Prof. Werner’s and Dr. Löhler’s letter again and ask you to return your replies by using the following link: https://de.research.net/s/...

Since the survey is anonymous, it is not possible for us to see if you already participated. In such a case please consider this reminder as irrelevant.

With best wishes for the weekend, 

Ulrike Fischer 

German Society of Oto-Rhino-Laryngology, Head and Neck Surgery 

President: Prof. Dr. med. Jochen A. Werner 

Friedrich-Wilhelm-Str. 2 

53113 Bonn 

Phone: +49-228-9239220 

Fax: +49-228-92392210 

info@hno.org 

http://www.hno.org/

## Notes

### Competing interests

The authors declare that they have no competing interests.

### Acknowledgements

The authors cordially thank all participating hospitals and practices for their commitment and the multiple, helpful replies. We also thank Dr. Maria Huber and Ralf Tostmann from the Study Center of Freiburg for their support in the whole study process. Special thanks to Alexander Hellmer of the Study Center of Freiburg for his excellent and patient support regarding statistics.

We also thank especially Ulrike Fischer from the German ENT Society in Bonn and Ilona Böhnke from the Professional Association of German Otolaryngologists for their excellent organization of the e-mail-based survey including all reminders.

## Figures and Tables

**Table 1:**
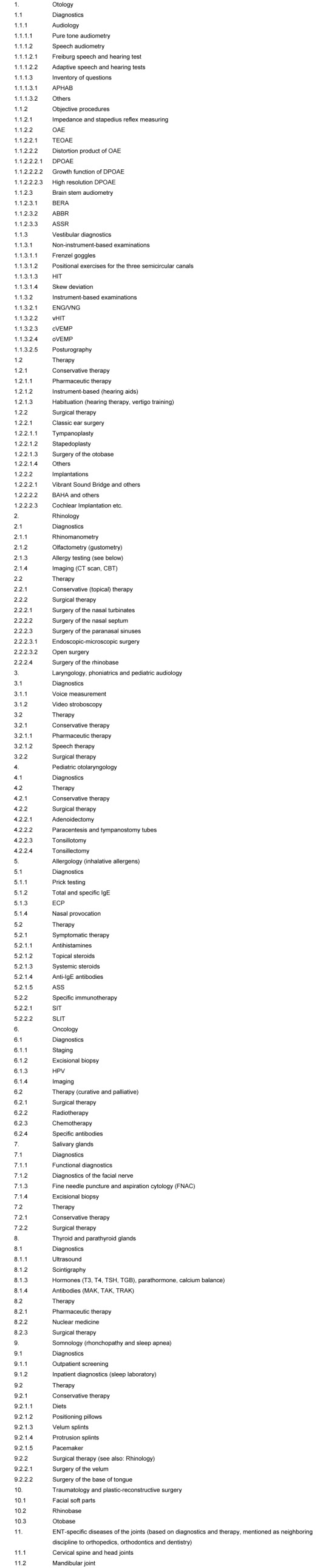
ENT-specific system for the closed survey (explanations can be found in the text)

**Table 2:**
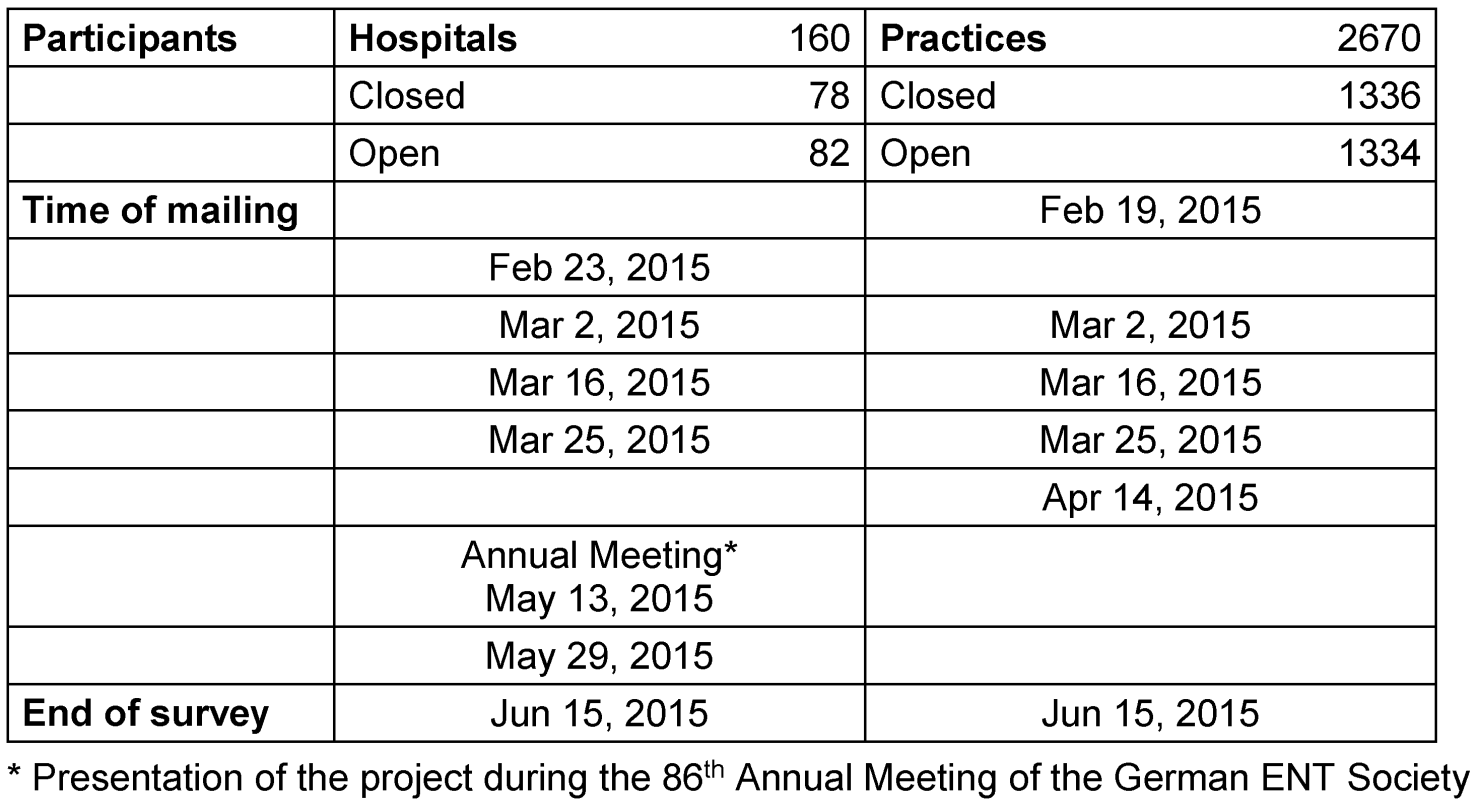
Process of the survey

**Table 3:**
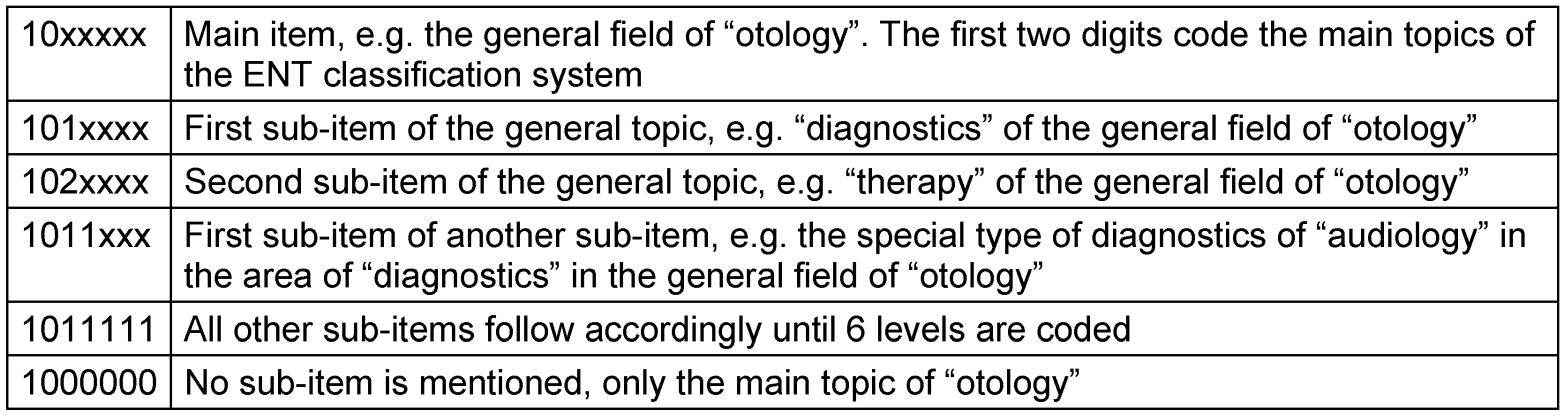
Coding scheme based on the example of question 1 of the closed survey of hospitals (explanations are found in the text)

**Table 4:**
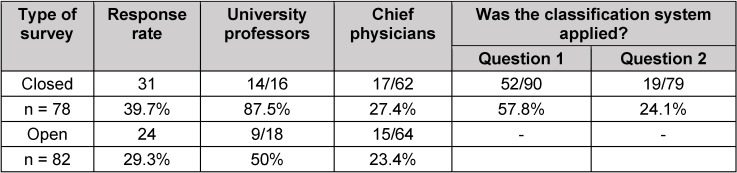
Response rate of hospitals

**Table 5:**
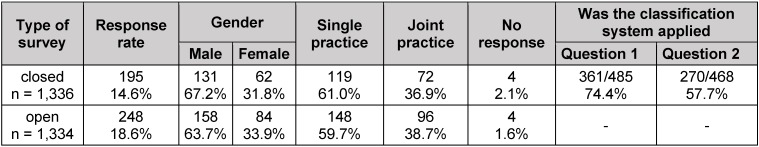
Basic data of practices

**Table 6:**
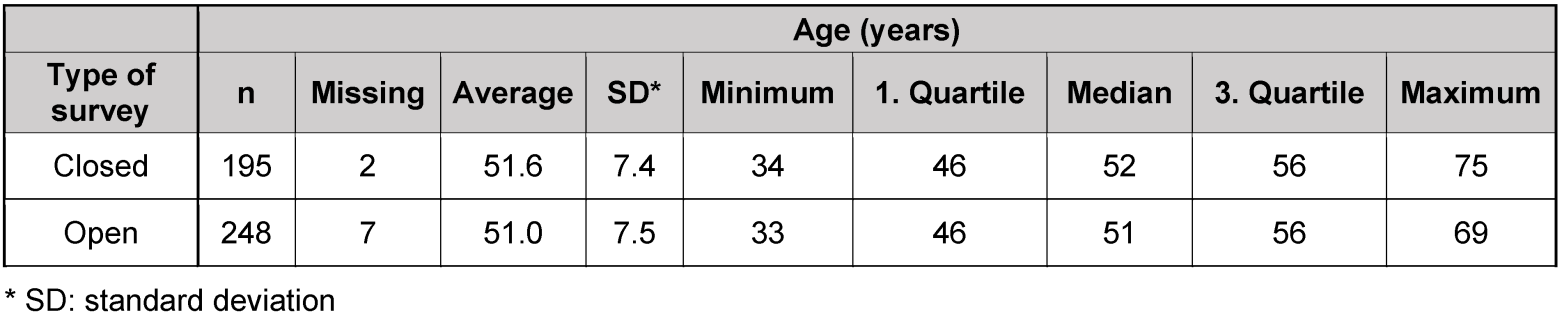
Age distribution of practices

**Table 7:**
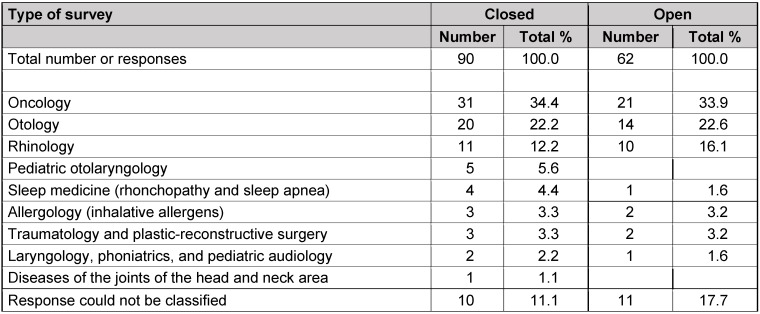
Results of question 1 (hospitals); fields with particular need of research

**Table 8:**
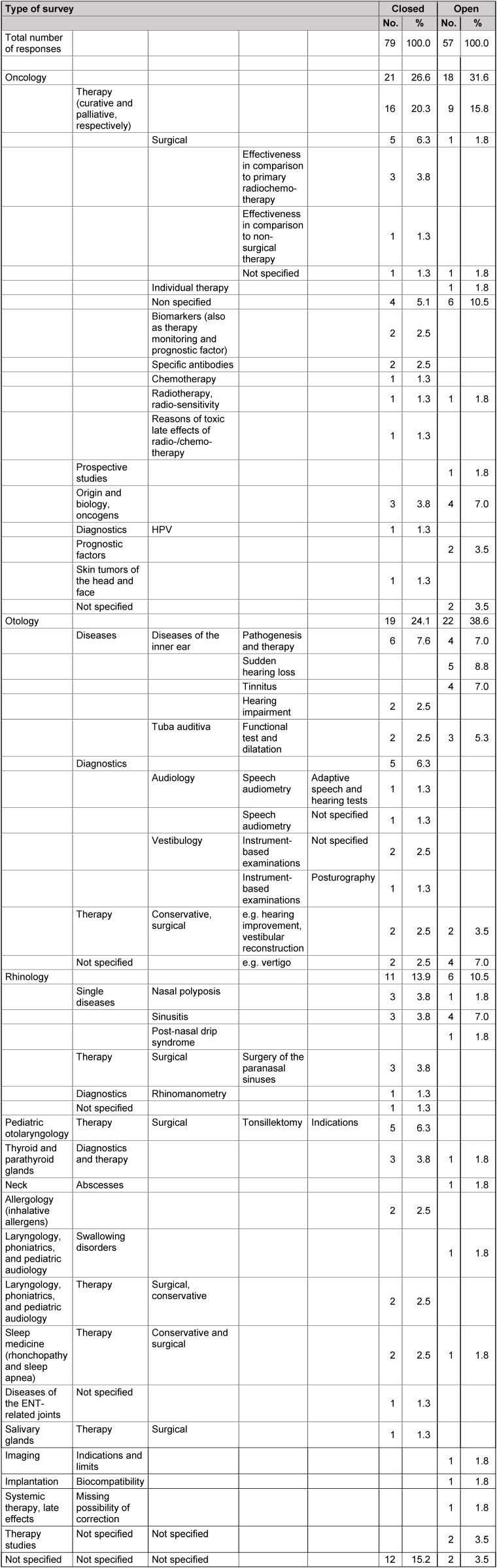
Results of question 2 (hospitals); currently relevant evidence gaps

**Table 9:**
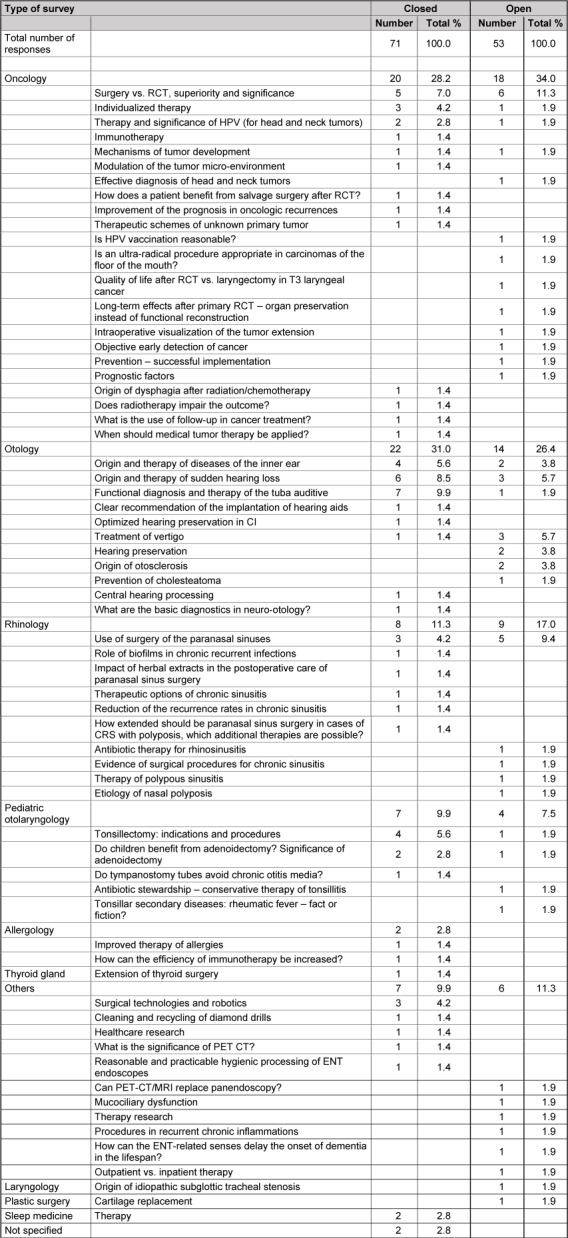
Responses of question 3 (hospitals); issues that require urgent answers

**Table 10:**
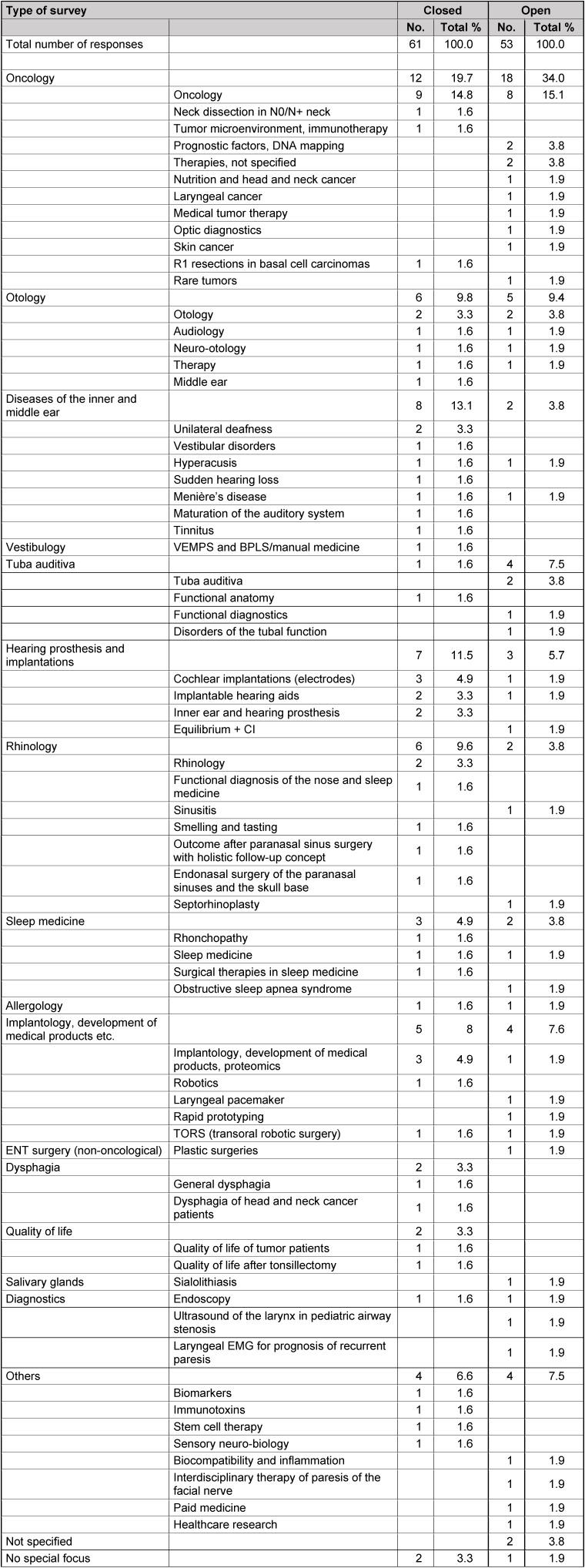
Research focus of hospitals

**Table 11:**
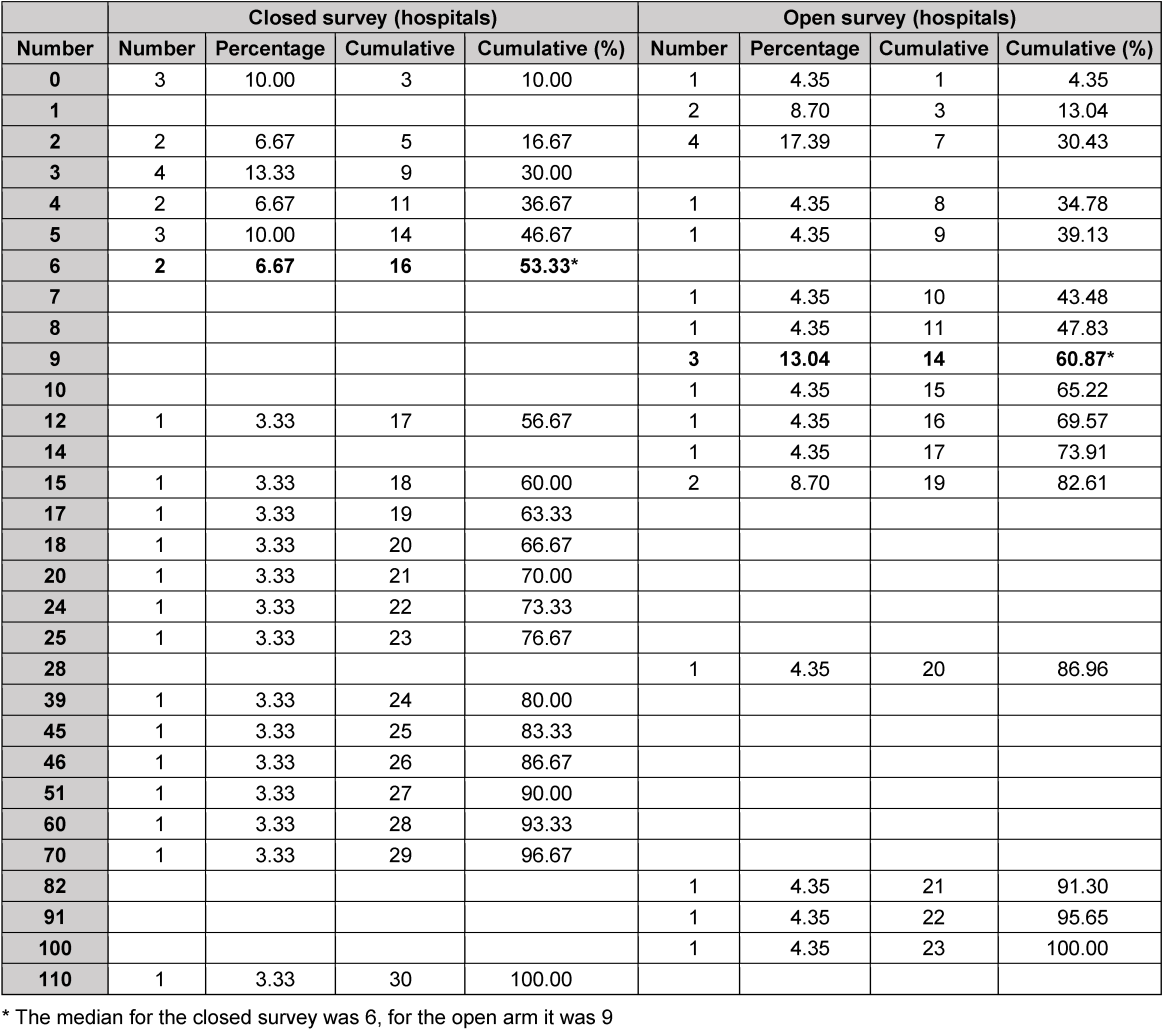
Number of publications (hospitals) from 2013 to 2014

**Table 12:**
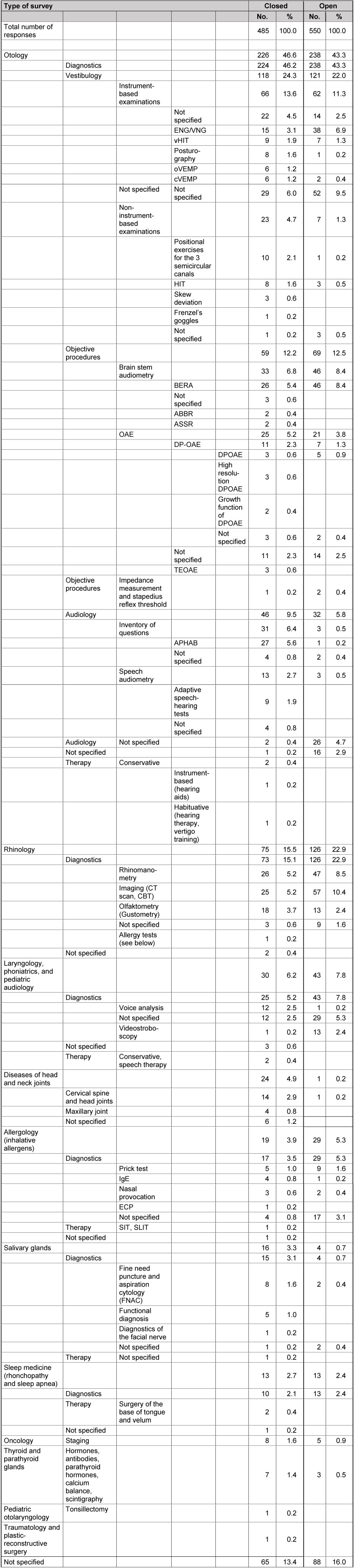
Results of question 1 (practices) regarding uncertainties of diagnostic procedures

**Table 13:**
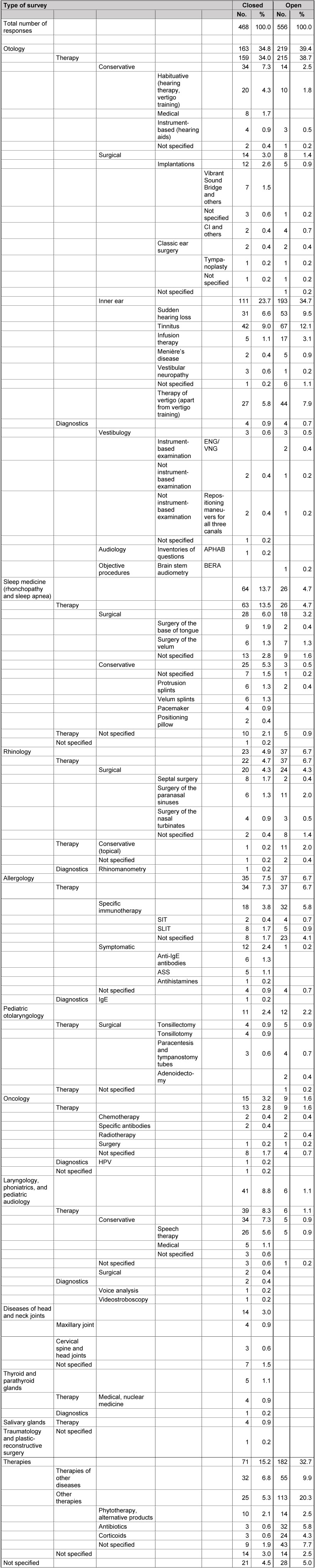
Results of question 2 (practices)

**Table 14:**
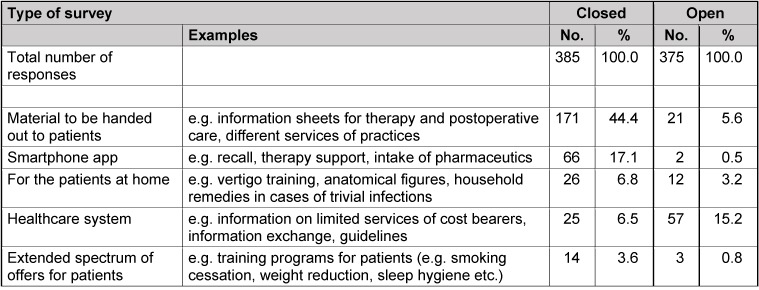
Results of question 3 (practices)
